# Motion-BIDS: an extension to the brain imaging data structure to organize motion data for reproducible research

**DOI:** 10.1038/s41597-024-03559-8

**Published:** 2024-07-02

**Authors:** Sein Jeung, Helena Cockx, Stefan Appelhoff, Timotheus Berg, Klaus Gramann, Sören Grothkopp, Elke Warmerdam, Clint Hansen, Robert Oostenveld, BIDS Maintainers, Stefan Appelhoff, Christopher J. Markiewicz, Taylor Salo, Rémi Gau, Ross Blair, Anthony Galassi, Eric Earl, Christine Rogers, Nell Hardcastle, Kimberly Ray, Julius Welzel

**Affiliations:** 1 Technical University of Berlin, Berlin, Germany; 2 Max Planck Institute for Human Cognitive and Brain Sciences, Leipzig, Germany; 3 Radboud University, Nijmegen, the Netherlands; 4 Donders Institute for Brain, Cognition and Behaviour, Nijmegen, the Netherlands; 5 Max Planck Institute for Human Development, Berlin, Germany; 6 Saarland University, Saarbrücken, Germany; 7 Kiel University, Kiel, Germany; 8 Karolinska Institutet, Stockholm, Sweden; 9 Department of Psychology, Stanford University, California, US; 10 University of Pennsylvania, Philadelphia, Pennsylvania US; 11 Origamin Lab, The Neuro, McGill University, Montreal, Quebec Canada; 12 Intramural Research Program, National Institute of Mental Health, Bethesda, MD USA; 13 McGill Centre for Integrative Neuroscience, Montreal Neurological Institute, Montreal, Canada; 14 University of Texas, Austin, USA

**Keywords:** Research management, Data publication and archiving

## Abstract

We present an extension to the Brain Imaging Data Structure (BIDS) for motion data. Motion data is frequently recorded alongside human brain imaging and electrophysiological data. The goal of Motion-BIDS is to make motion data interoperable across different laboratories and with other data modalities in human brain and behavioral research. To this end, Motion-BIDS standardizes the data format and metadata structure. It describes how to document experimental details, considering the diversity of hardware and software systems for motion data. This promotes findable, accessible, interoperable, and reusable data sharing and Open Science in human motion research.

## Introduction

### Importance of motion data in human neuro- and behavioral science

In the 1830s, the Weber brothers were among the first to report detailed information about temporal and spatial parameters of locomotion of different body parts^1^. Since then, advances in recording technology have led motion tracking to cover a wide range of applications. In the entertainment industry, motion is recorded to create realistic animation in films and games. In immersive virtual reality (VR) systems, motion data is used for interaction between users and the simulated environment. The motion of human body parts is the subject of study in the field of biomechanics and is a relevant source of information in numerous other research areas, such as medicine, sports science, ergonomics, and neuroscience.

In human behavioral research, biomechanical features extracted from motion data, such as gait patterns, provide insights into underlying cognitive processes and have diagnostic value^2^. For example, step length is related to the severity of Parkinson’s disease^3^. Cognitive impairment in older adults is associated with gait slowing and the prevalence of falls^4^. Mobile brain-body imaging^5,6^ studies record and analyze motion data together with neuroimaging data, typically electroencephalogram (EEG)^7,8^ and functional near-infrared spectroscopy (fNIRS)^9^. Recent technological advances allow invasive recording of electrophysiological data while the participant is in motion^10,11^, further increasing the importance of motion data in human neuroscience^12^.

Motion of non-physical (virtual) objects can serve as the proxy for certain cognitive-behavioral processes. A typical example is the motion of a virtual first-person perspective camera, controlled by a participant, comparable to how they would move through the physical space themselves. This type of motion data has the advantage of being compatible with more strictly stationary neuroimaging setups, such as functional magnetic resonance imaging (fMRI) experiments^13,14^. For instance, virtual heading and velocity simultaneously recorded with the fMRI data were successfully used to identify locomotion-modulated activity of the entorhinal cortex in human participants^15^.

### Overview of various motion tracking systems

Motivated by the widespread use of motion data in research on human cognition and behavior, we present Motion-BIDS as a standard for organizing motion (meta) data in neuro- and behavioral science, building on the Brain Imaging Data Structure (BIDS)^16^. Motion-BIDS is compatible with a wide range of motion tracking systems. Therefore, the term “motion tracking” in this context is used in a broad sense, encompassing the recording of the movement of an object in physical space, commonly referred to as “motion capture” or “pose estimation”, as well as in virtual space. Below, we provide a summary of various motion tracking systems used in research.

Optical motion tracking systems use cameras either with body-attached markers (which can be passive, for example, Vicon; or active, such as Optotrak 3020) or without markers (for example, OpenPose^17^ or FreeMoCap^18^) to track the movement of body parts. Other systems use electromagnetic transmitters or sensors (for example, Polhemus) to track movement. Motion tracking systems based on inertial measurement units (IMUs) measure acceleration and angular velocity (for example, Xsens), often together with magnetic field strength. Wearable global positioning system (GPS) trackers, which record the movement in respect to a global navigation satellite system, may be used in combination with such systems^19^. In addition to the physical motion of body parts, tasks presented in virtual space may simulate the experience of moving through physical space. The resulting data can be represented in the same format as the motion data through the physical world. For instance, the positions of a virtual camera can be described as Cartesian coordinates along the virtual spatial axes. For further reading, see Klette and Tee^20^ on history of human motion capture, Colyer *et al*.^21^ and Desmaris *et al*.^22^ on markerless motion tracking, Menolotto *et al*.^23^ on industrial applications, Majumder *et al*.^24^ on motion tracking in remote health, and van der Kruk and Reijne^25^ on sports science applications.

The diversity of motion tracking systems results in heterogeneous types of data and various temporal resolutions, ranging from 1 Hz with GPS up to over 2000 Hz^25^. The systems typically store the data in proprietary device-specific file formats^25,26^. Consequently, the same type of data may have non-unique representations. For example, orientations can be represented as either quaternions or Euler angles. Additionally, the recording system may apply different levels of preprocessing. For instance, joint angles may be inferred from marker positions and directly stored in the recording output.

Such diversity in output formats requires a standard for data management and sharing to prioritize the types of motion data and metadata that are most relevant in human neuro- and behavioral science. Restricting how these fundamental aspects should be communicated improves interoperability of motion data. At the same time, it should still allow documentation of idiosyncrasies of the recording systems. The contribution of Motion-BIDS to this end is threefold. (i) It provides a flexible way to define what is construed as a single motion tracking system, agnostic to the type of recording technology used. (ii) It requires users to share metadata that are the most central to interpretation of motion data. (iii) By embedding motion data in the BIDS framework, it supports the growing junction between biomechanics and human neuro- and behavioral science. Concretely, it facilitates the management of motion data together with other data modalities in a harmonized and time-synchronized manner. The next paragraphs provide a brief overview of BIDS.

### The Brain Imaging Data Structure

The community-driven BIDS initiative has been successful in organizing, documenting and contributing to modern-day FAIR (findable, accessible, interoperable, and reusable) data management and sharing^27^. BIDS was developed for managing and sharing data collected using neuroimaging methods, including (functional) MRI^16^, magnetoencephalography^28^ (MEG), EEG^29^, positron emission tomography^30^, microscopy^31^, and NIRS^32^. The extensions to the original BIDS-specification for MRI were driven by the respective communities of researchers. The Motion-BIDS extension to BIDS was established with involvement of researchers from the neuroscience and biomechanics community, under the guidance of the BIDS steering committee. By providing a standard for the organization of data and metadata, BIDS aims to promote reproducibility of findings, data reusability, and general research efficiency. Broadly, BIDS specifies (i) which data to include in a data set, (ii) how to organize it over directories and files, (iii) the naming of files, and importantly (iv) the organization and format of metadata to complement the data.

Adhering to the framework of BIDS, Motion-BIDS focuses on the most central characteristics of motion data that are relevant for their use within the context of human cognitive-behavioral research. We aim to document all data and metadata that is needed for the correct interpretation and (re)use of the data. We encourage idiosyncratic features of different recording systems to be documented as metadata. This manuscript highlights the most important aspects where Motion-BIDS extends earlier versions of the BIDS standard. The main BIDS website (https://bids.neuroimaging.io/specification.html) links to the full documentation of the Motion-BIDS extension (https://bids-specification.readthedocs.io/en/stable/modality-specific-files/motion.html).

### Scope of Motion-BIDS

Motion-BIDS is designed primarily for sharing motion data as a time series of position or orientation samples associated with physical and virtual space. The first and second temporal derivatives (for example, acceleration and angular velocity in IMU-based systems) are included in the scope. Other common data that changes over time, such as joint angles and magnetic field strengths, are also part of the specification. Non-continuous motion data (for example, timing of heel-strikes during walking) falls out of the scope of the present proposal but, when temporally related to the data, can be stored as events.

The Motion-BIDS extension describes how raw data and related metadata is to be represented and documented. Data that results from offline processing and analysis based on assumptions specific to the research question using software other than that of the acquisition system is referred to as derived data. Storing derived data is not yet standardized in Motion-BIDS.

Typically, the files in the proprietary format output by the original acquisition software differ from BIDS-formatted data. These files are referred to as source data in the BIDS standard and often need to be converted to raw data in order to comply with BIDS. The source data can optionally be stored and shared in the “sourcedata” directory. Nearly all motion tracking systems apply some level of online processing during acquisition (for example, identification of reflective markers in a camera-based tracking, or fusion of data from multiple IMU components). Yet, data that is thus processed by the acquisition system is still defined in BIDS as source data. This is similar to how MRI source data is defined in BIDS: the images before offline processing result from online processing of the signals recorded by the MRI scanner.

### Motion-specific extensions to BIDS

Motion-BIDS provides users with a method to flexibly group data channels, describe the space in which data is to be interpreted, and preserve the temporal synchrony with data from other modalities or with events.

### General principles and folder hierarchy

**Fig. 1:**
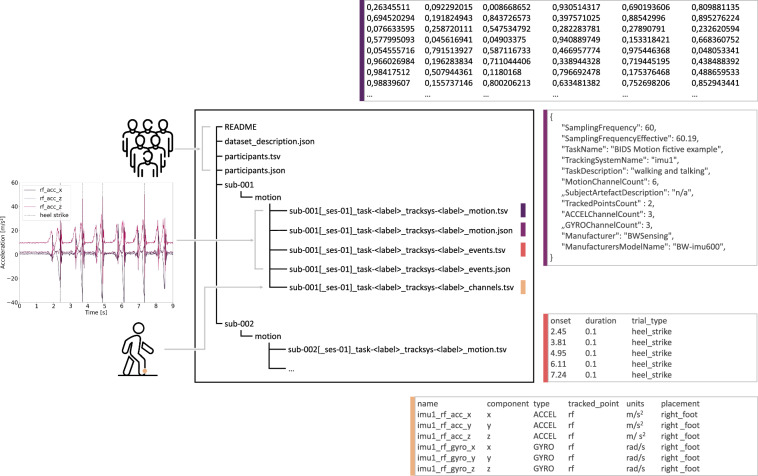
Overview of an exemplary Motion-BIDS data set that contains three accelerometer channels and three gyroscope channels along with heel strike events. On the left, three-channel accelerometer data and the timing of heel strike events are visualized. In the middle, the directory structure following BIDS is shown. Each subject-specific folder (for example, “sub-001”) contains a modality-specific motion folder, within which “motion.tsv”, “motion.json”, “events.tsv”, and “channels.tsv” files are found. Exemplary contents for each file type are shown in boxes around the central panel.

In accordance with the BIDS standards, the motion data for each subject is stored in a modality-specific subdirectory within the subject or session folder. In the following sections, we describe and explain the contents of this directory. See Fig. 1 for overview.

In Motion-BIDS, the motion data is stored as a tab-separated values (TSV) file. The file MUST be shared in a subdirectory “motion” (see Figure 1) and is named “sub-XX_task-YY_tracksys-ZZ_motion.tsv”. In each “motion” folder, data and metadata from at least one tracking system are stored.

### Tracking system

We define a tracking system as a group of channels that synchronously sample motion data from one or multiple tracked points. To be grouped as a single tracking system, channels MUST share the core parameters of sampling (namely the sampling rate and the duration) as well as hardware and software properties, resulting in the same number of samples and, if available, a single latency channel associated with the rest of the channels. The matching number of samples between channels is a prerequisite for the multichannel data to be saved in one TSV file, thus making it a single tracking system. For example, a tracking system recording the positions and orientations of the left wrist of a participant in 3D would consist of seven channels: three for positions, three for orientations as Euler angles, and one latency channel containing the latency values shared across the position and orientation samples.

Such a tracking system may comprise multiple physical devices. For example, an optical system tracking multiple markers or the base station from a motion tracking system that synchronizes the wireless input from multiple IMUs may be grouped as a single tracking system. On the other hand, if multiple components within the recording setup are not synchronized by the manufacturer’s hard- or software and have different numbers of samples per device, they are to be defined as separate tracking systems, stored in separate TSV files.

We introduce the new BIDS entity “tracksys” (for tracking system) as part of the file name. This enables the data from each tracking system to be uniquely represented as a single TSV file in which each column corresponds to one channel and each row corresponds to one time point.

#### Files per tracking system

1. The required “sub-XX_task-YY_tracksys-ZZ_motion.tsv” file contains the raw time series data. Each column represents one channel, each row represents samples collected at a single time point. As no headers are included in a “motion.tsv” file, the ordering of the columns in the TSV file is required to match the ordering in the corresponding “sub-XX_task-YY_tracksys-ZZ_channels.tsv” file.
2. The required “sub-XX_task-YY_tracksys-ZZ_motion.json” file holds metadata to that specific tracking system such as the sampling rate (required), manufacturer and model, and channel count (recommended) in addition to generic information which may be shared with other modalities (see Table 1 for details).

**Table 1:** Overview of motion specific fields for metadata in *_tracksys-<label>_motion.json file.

Key name	Requirement level	Data type
SamplingFrequency	REQUIRED	number
ACCELChannelCount	RECOMMENDED	number
ANGACCChannelCount	RECOMMENDED	number
GYROChannelCount	RECOMMENDED	number
JNTANGChannelCount	RECOMMENDED	number
LATENCYChannelCount	RECOMMENDED	number
MAGNChannelCount	RECOMMENDED	number
MISCChannelCount	RECOMMENDED	number
MissingValues	RECOMMENDED	string
MotionChannelCount	RECOMMENDED	number
ORNTChannelCount	RECOMMENDED	number
POSChannelCount	RECOMMENDED	number
SamplingFrequencyEffective	RECOMMENDED	number
SubjectArtefactDescription	RECOMMENDED	string
TrackedPointsCount	RECOMMENDED	number
VELChannelCount	RECOMMENDED	number
TrackingSystemName	OPTIONAL	string
3. The required “sub-XX_task-YY_tracksys-ZZ_channels.tsv” file contains one row per recorded channel.
  The “channels.tsv” provides the name for each channel, plus metadata, including the spatial component (required), channel type (required), and the user-defined name of the point that is being tracked (recommended). The labels for reference frames (recommended) can be specified within this file for mapping between data channels and the description of corresponding reference frames (see Table 2 for details).

**Table 2:** Overview of columns for metadata in *_tracksys-<label>_channels.tsv file.

Column name	Requirement Level	Data type
name	REQUIRED	string
component	REQUIRED	string
type	REQUIRED	string
tracked_point	REQUIRED	string
units	REQUIRED	string
placement	RECOMMENDED	string
reference_frame	RECOMMENDED	string
description	OPTIONAL	string
sampling_frequency	OPTIONAL	number
status	OPTIONAL	string
status_description	OPTIONAL	string
4. The optional “sub-XX_task-YY_tracksys-ZZ_channels.json” file can complement the “channels.tsv” file. In Motion-BIDS, this file can be used to describe the spatial properties of the reference frames used in the data set.
5. The optional “sub-XX_task-YY[_tracksys-ZZ]_events.tsv” file contains events data. If there are multiple tracking systems and multiple “motion.tsv” files in the directory, including “tracksys-ZZ” entity in the file name is recommended.
6. The optional “sub-XX_task-YY[_tracksys-ZZ]_events.json” file is recommended to complement the “events.tsv” file with a data dictionary for events.

#### Description of the position and orientation channels

Motion-BIDS prescribes the use of Cartesian coordinates for representation of positions and their time derivatives. Data recorded using other coordinate systems (for example, the polar coordinate system) MUST be converted to Cartesian coordinates. The Cartesian coordinate system in Motion-BIDS consists of up to 3 components, x, y, and z, each of which represents the position of the perpendicular projection of a point onto the corresponding axis, expressed as the signed distance from the origin. The component (x, y, or z) associated with each data channel is stored in the column “component” in the “channels.tsv” file. A full description of spatial relations between the axes can be provided by specifying the recommended column “reference_frame” in the “channels.tsv” with an accompanying “channels.json” file. See the paragraphs “Description of reference frames” below for further details.

Motion-BIDS requires orientations and their temporal derivatives to be represented as Euler angles or quaternions. Euler angles represent 3D orientations as a sequence of elemental rotations around the spatial axes. Like position channels, the components (x, y, or z) associated with orientation channels are stored in the column “component” in the “channels.tsv” file. These rotations are non-commutative, meaning that the result depends on the order in which the operations are performed. Therefore, Motion-BIDS recommends the specification of the order (for example, XYZ or YZX) of elemental rotations whenever 3D orientation data is shared. For details regarding how this information is represented, see the paragraphs “Description of reference frames” below.

An alternative representation for orientations in 3D and their time derivatives is quaternions. A quaternion consists of four numbers, three corresponding to the three spatial axes and one non-axial component. This representation is used in computer graphics and robotics, due to its practical advantages. Unlike Euler angles, quaternions do not face the problem of gimbal lock, which is when a degree of freedom is lost at certain orientations in a system represented by Euler angles, hindering further rotation around one of the axes^33^. Virtual motion tracking systems often output orientation time series in quaternions. For sharing quaternions in Motion-BIDS, the channels MUST be labeled as “quat_x”, “quat_y”, “quat_z” for components corresponding to the three spatial axes and “quat_w” for the non-axial component. Erroneous conversion from quaternions to Euler angles is difficult to reverse and may create a barrier for data sharing. Thus, it is recommended that orientation time series recorded in quaternions preserve the representation in BIDS. However, relevant details regarding the conversion steps, as well as the direction of the rotations (left-hand versus right-hand), can optionally be shared as detailed in the paragraphs “Description of reference frames” below.

#### Description of reference frames

Meaningful interpretation of the time-series of positions and/or orientations requires an adequate description of the spatial axes. Motion-BIDS specifies how to describe the spatial axes so that “anterior-posterior (forward-backward)”, “left-right”, and “superior-inferior (up-down)” can be associated with the data. In Motion-BIDS, the spatial axes are not necessarily bound to body parts but may extend beyond peripersonal space, relating to other reference frames such as the lab space. This is to be distinguished from coordinate systems defined in BIDS for static images (MRI) or sensor (EEG, MEG) positions, which have anatomical connotations.

Motion-BIDS RECOMMENDS the use of the “reference_frame” column in the “channels.tsv” file. An entry to this column is a user-defined string denoting the reference frame associated with relevant channels. For instance, the keyword “global” can be chosen to refer to the lab space, and “local” to refer to the participant’s body as a moving origin. Once mapped to relevant channels, each reference frame can be described in the “channels.json” file. A “channels.json” MAY have a field “reference_frame”, which SHOULD contain a “Levels” field listing the names of reference frames (“global” or “local” for example) as objects. Each object has three RECOMMENDED fields: “SpatialAxes”, “RotationRule”, and “RotationOrder”, and one OPTIONAL field “Description”. All channels associated with a single reference frame are assumed to share all of the properties represented in these four fields. As many reference frames can be defined as the number of channel groups that differ from each other in terms of their spatial properties listed here.

The entry to field “SpatialAxes” describes spatial axes, the lines with respect to which a position or an orientation is defined. They are denoted by uppercase letters X, Y, and Z. Motion-BIDS supports the description of up to three spatial axes given by strings consisting of three characters, each of which indicates the spatial orientation and positive direction of the corresponding axis. The description is a sequence of three characters: A or P (anterior-posterior), L or R (left-right), and S or I (superior-inferior). The position of a character in the sequence determines which of the X, Y, or Z axes it maps to. For example, “ARI” for X-anterior, Y-right, Z-inferior. For 1D or 2D cases, only specify the used axes and use the character “_” for unused axes (“A_R” when the Y axis is not used, for instance).

The “RotationRule” field contains information about the handedness of rotations represented in orientation channels. The handedness of the rotation (left-hand versus right-hand) determines the positive direction of rotation with respect to the spatial axis. The left-hand rule for rotation expresses a clockwise rotation as a positive angle when viewed from the positive end of the axis about which the rotation is applied. The right-hand rule is defined by the same principle with the rotation being counterclockwise instead. Making a fist with one’s left or right hand while holding the thumb straight is a helpful way to visualize the difference between the two rotation rules, with the curled fingertips directing to the positive angular direction, and the tip of the thumb pointing to what corresponds to the positive end of the axis. The direction of rotation around each axis MUST be specified as “left-hand” or “right-hand”.

The “RotationOrder” field specifies the order of the three elemental rotations to be applied in 3D orientation data. Due to the non-commutative property of elemental rotations that make up Euler angles, applying the rotations in a wrong order can output a wrong orientation. Another decisive parameter for the interpretation of Euler angles is whether the elemental rotations are extrinsic or intrinsic. An extrinsic rotation uses the original axis as the fixed reference, while an intrinsic rotation uses the axis resulting from the previous rotation as the new reference. In Motion-BIDS, the rotation order is specified as a sequence of extrinsic rotations. Furthermore, the rotations are to be defined around three distinct axes (so-called Tait-Bryan angles, for example, XYZ), and not as classic Euler angles about two axes (for example, XYX). Designated keywords for expressing rotation order correspond to the six permutations of the three axes: XYZ, XZY, YXZ, YZX, ZXY, or ZYX.

Finally, the optional field “Description” MAY contain a freeform text description about the reference frame, potentially referring to a definition of the reference frame outside of BIDS.

### Synchronization and the latency channel

Motion-BIDS allows the recordings from multiple systems to be time-synchronized. This applies both to multiple motion tracking systems that are used simultaneously and to the combination of motion tracking with other data modalities, such as EEG. When simultaneously recorded data files have different temporal characteristics (for example, motion at 80 Hz and EEG at 1000 Hz), it is useful to provide information needed for temporal alignment with the highest precision possible.

Motion-BIDS introduces a latency channel that contains timing information per sample relative to the recording onset. Recording the latency of each sample is particularly useful when the temporal intervals between consecutive samples are not uniform. This translates to fluctuating sampling frequency throughout the duration of a continuous recording. This jitter can accumulate over time and cause the temporal alignment based on sampling frequency to be time-shifted relative to other recordings. For tracking systems that do not provide single-sample timestamp information, the latency of each sample can be reconstructed based on the effective sampling frequency (recommended field “SamplingFrequencyEffective”), if available, or the nominal sampling frequency (required field “SamplingFrequency”), both found in the “motion.json” metadata file.

Synchronizing the onset of motion data between different tracking systems and/or with data from other modalities is achieved using the “sub-XX_scans.tsv” file, which contains an optional column “acq_time” that documents the onset of acquisition in the datetime format designated by BIDS.

The “events.tsv” files alongside each modality document the task-relevant events and their timings. If multiple tracking systems are used in a recording session, the “events.tsv” file(s) SHOULD contain a “tracksys” entity for unambiguous mapping between the events and data files. For example, if the data was collected using a combination of an optical motion capture system and an IMU-based system, the “sub-XX_task-YY_tracksys-optical_events.tsv” and the “sub-XX_task-YY_tracksys-IMU_events.tsv” files may both be present. The event onsets would be expressed in each “events.tsv” file relative to the onset of the corresponding recording file.

### Example Motion-BIDS data sets

Four example data sets that are formatted using the Motion-BIDS standards are described below, three of which are in the BIDS-examples GitHub repository (https://github.com/bids-standard/bids-examples). These versions of the data sets contain empty raw data files, as the goal of the repository is to serve as an example of how to structure the data sets and to support lightweight software tests. The full version of data set 1 is found on OpenNeuro^34^ and part of data set 3 on Open Science Framework^35^. The fourth example data set can be found on the Radboud Data Repository.

1. The Spot Rotation data set^36^ (“motion_spotrotation”) is an example of multiple tracking systems combining virtual as well as physical motion data. The full data set is available on OpenNeuro (https://openneuro.org/datasets/ds004460/versions/1.1.0)^37^. In one session, participants rotated their heads while wearing a head-mounted display for immersive VR. The head motion was tracked using both the tracking system integrated in the VR setup and an optical motion tracking system. In another session, participants used a joystick to simulate rotation and the virtual orientation time series of the camera was recorded. All sessions include concurrently recorded EEG data.
2. The Walking Old and Young data set (“motion_dualtask”) shows an example of optical marker-based motion tracking systems recording multiple body parts. Participants were walking in straight lines through physical space while performing a cognitive task. Here motion data recorded from different body parts differed from each other in terms of their temporal profiles, resulting in as many “tracking systems” or “motion.tsv” files as the number of body parts tracked. All sessions include concurrently recorded EEG data.
3. The dual system validation data set^38^ (“motion_systemvaildation”) contains data from healthy participants and individuals with neurological disorders, including Parkinson’s disease, stroke, multiple sclerosis, and chronic lower back pain. Full data files from two participants are available on Open Science Framework (https://osf.io/n64ga/)^39^. Full body motion measurements were taken of 167 subjects using a Noraxon IMU system and a Qualisys optical motion capture system. The participants undertook various standardized mobility tests and everyday activities.
4. The fNIRS-gait data set^40^ (10.34973/k7ce-6n58) contains full-body motion capture data from an Xsens system consisting of 17 IMUs, recorded in 22 individuals with Parkinson’s disease and 22 control participants, along with time-synchronized fNIRS data. Participants performed a gait task including 180-degree turns, passing through a narrow doorway, stopping, and starting. The files in this data set are converted and exported from their original .mvnx format of the MVN Awinda software (version 2020.0.1) and contain acceleration, angular velocity and magnetometer data of each sensor, as well as positions and orientations of all body parts. Additionally, center of mass position, joint angles, and foot contact data are shared.

## Discussion

Motion-BIDS focuses on addressing the most fundamental aspects of motion data, rather than providing a fine-tuned solution for each type of recording system. Through the prescription of metadata fields and a common data format, BIDS-Motion enhances the interoperability of motion data sets and ultimately the reproducibility of research using motion data.

By using shared definitions of channels regardless of the type of motion tracking system in use, the development of software tools agnostic to the exact type of recording system is made easier. Open source toolboxes such as EEGLAB^41^, FieldTrip^42^, and MNE-Python^43^ for analysis of EEG data, frequently used together with motion capture, support conversion, organization, and importing of BIDS-formatted motion data for joint analysis with brain recordings.

Prioritizing the ease of sharing and interpreting the data naturally results in a number of persisting challenges such as the lack of the means to share the precise sensor placement and detailed definitions of spatial axes within the BIDS framework. This reflects the lack of field consensus on whether and how the complexity of such spatial quantities can be universally communicated. Motion-BIDS considers potential solutions for these issues and aims to be compatible with ongoing or upcoming efforts coevolving with BIDS.

Many methods for motion tracking of human participants use passive markers, active sensors, or transmitters that are placed on various body parts and with various orientations. For some systems, placement and orientation standards are defined by manufacturers and used consistently across multiple data sets acquired with the same equipment. Standards for nomenclature of body parts and anatomical landmarks are common in the (para)medical field and used in movement science^44,45^. Motion-BIDS does not designate any nomenclature for documenting this information.

Depending on the type of motion tracking system and the processing applied by the recording software, motion data can be expressed with respect to various interrelated reference frames. For example, the reference frame can be a physical lab space with the origin of the coordinate system being the center of the floor. A local reference frame can be nested within this lab space reference frame. In the current version of Motion-BIDS, it is not explicitly prescribed how such relations should be represented.

Motion-BIDS is neither the first nor the only attempt to establish standards on motion data in research. One noteworthy body of work is the reporting guidelines published by the International Society of Biomechanics (ISB) standardization committee^46^. The overlap between the ISB guidelines and Motion-BIDS lies in the focus on providing uniformity in the shared data, regardless of how the data was acquired. However, the two standards solve distinct sets of problems in distinct contexts. Firstly, the ISB guidelines apply to how the results are to be *reported* in publications, whereas BIDS is for *data sharing* and management. In addition, the ISB guidelines are presented as recommendations, while BIDS explicitly distinguishes between varying levels of restriction (such as “required”, “recommended”, “optional”). Furthermore, the ISB guidelines are developed mainly for advanced biomechanics research, resulting in high-resolution definition of reference frames and body parts. Individual body parts, for example the feet^47^, can have multiple subsegments defined by the recommendations of ISB. On the other hand, Motion-BIDS does not yet prescribe the way the precise localisation of relevant body parts are to be described and instead prioritizes the ease of sharing and reusing the data in the context of neuro- and behavioral science.

The metadata fields in the current version of Motion-BIDS serve as placeholders for potentially linking the specification to an external library. Motion-BIDS will develop in close coordination with other relevant efforts such as the ISB standards and hierarchical event descriptors^48^ (HED). In HED, anatomy and spatial relations commonly appearing in human neuro- and behavioral science are listed. A recent initiative, EUROBENCH^49^, has undertaken an interdisciplinary approach to harmonize motion data, streamlining benchmarking methods for robotic and human bipedal motion. This effort reflects a concerted attempt to enhance the standardization and comparability of motion data across diverse applications.
